# Molecular allergen profiling in horses by microarray reveals Fag e 2 from buckwheat as a frequent sensitizer

**DOI:** 10.1111/all.13417

**Published:** 2018-02-27

**Authors:** L. Einhorn, G. Hofstetter, S. Brandt, E. K. Hainisch, I. Fukuda, K. Kusano, A. Scheynius, I. Mittermann, Y. Resch‐Marat, S. Vrtala, R. Valenta, E. Marti, C. Rhyner, R. Crameri, R. Satoh, R. Teshima, A. Tanaka, H. Sato, H. Matsuda, I. Pali‐Schöll, E. Jensen‐Jarolim

**Affiliations:** ^1^ The interuniversity Messerli Research Institute University of Veterinary Medicine Vienna Medical University Vienna and University Vienna Vienna Austria; ^2^ Institute of Pathophysiology and Allergy Research Center for Pathophysiology, Infectiology and Immunology Medical University of Vienna Vienna Austria; ^3^ Research Group Oncology Equine Clinic University of Veterinary Medicine Vienna Vienna Austria; ^4^ Racehorse Hospital Miho Training Center Japan Racing Association Mikoma Japan; ^5^ Science for Life Laboratory Department of Clinical Science and Education Karolinska Institutet, and Sachs’ Children and Youth Hospital Södersjukhuset Stockholm Sweden; ^6^ Department of Clinical Research and Veterinary Public Health Vetsuisse Faculty University of Bern Bern Switzerland; ^7^ Swiss Institute for Allergy and Asthma Research (SIAF) Davos Switzerland; ^8^ Division of Food Function Research Food Research Institute National Agriculture and Food Research Organization Tsukuba Japan; ^9^ National Institute of Health Sciences Tokyo Japan; ^10^ Laboratory of Comparative Animal Medicine Division of Animal Life Science Tokyo University of Agriculture and Technology Fuchu Japan; ^11^ Laboratory of Veterinary Molecular Pathology and Therapeutics Division of Animal Life Science Tokyo University of Agriculture and Technology Fuchu Japan; ^12^ AllergyCare Allergy Diagnosis and Study Center Vienna Austria

**Keywords:** allergen, microarray, component‐resolved diagnosis, horse, IgE, ISAC, molecular

## Abstract

**Background:**

Companion animals are also affected by IgE‐mediated allergies, but the eliciting molecules are largely unknown. We aimed at refining an allergen microarray to explore sensitization in horses and compare it to the human IgE reactivity profiles.

**Methods:**

Custom‐designed allergen microarray was produced on the basis of the ImmunoCAP ISAC technology containing 131 allergens. Sera from 51 horses derived from Europe or Japan were tested for specific IgE reactivity. The included horse patients were diagnosed for eczema due to insect bite hypersensitivity, chronic coughing, recurrent airway obstruction and urticaria or were clinically asymptomatic.

**Results:**

Horses showed individual IgE‐binding patterns irrespective of their health status, indicating sensitization. In contrast to European and Japanese human sensitization patterns, frequently recognized allergens were Aln g 1 from alder and Cyn d 1 from Bermuda grass, likely due to specific respiratory exposure around paddocks and near the ground. The most prevalent allergen for 72.5% of the tested horses (37/51) was the 2S‐albumin Fag e 2 from buckwheat, which recently gained importance not only in human but also in horse diet.

**Conclusion:**

In line with the One Health concept, covering human health, animal health and environmental health, allergen microarrays provide novel information on the allergen sensitization patterns of the companion animals around us, which may form a basis for allergen‐specific preventive and therapeutic concepts.

**Abbreviations**
BSA: bovine serum albumin
CAP: caponized
Cy3: cyanine 3 fluorophore
HDM: house dust mite
H: hour
HRP: horse radish peroxidase
ISAC131: ISAC, custom‐designed with 131 allergen molecules
ISAC: immuno‐solid‐phase allergen chip
ISU‐E: ISAC standardized units for IgE
ISU: ISAC standardized units
Min: minute
RAO: recurrent airway obstruction
rFag e 2: recombinant Fag e 2
RT: room temperature
SDS‐PAGE: sodium dodecyl sulphate–polyacrylamide gel electrophoresis
TBST: Tris‐buffered saline with Tween 20

## 1 INTRODUCTION

Allergies also affect domestic animals, such as dogs, cats and horses.^1^ The same allergen sources as in human allergy may be relevant for animals, such as pollen^2^ or food allergens.^3^ The importance of comparing human and animal allergic diseases and causative allergens has recently been acknowledged by the establishment of an interest group for Comparative and Veterinary Allergology in the European Academy of Allergy and Clinical Immunology (EAACI). In fact, comparative allergology ideally fits into the “One Health Concept,” which relies on three main pillars: human health, animal health and environmental health.^4^

In horses, allergen hypersensitivity^5^ leads to cutaneous symptoms, that is eczema^6^ or urticaria,^7^ and/or to respiratory symptoms in form of chronic coughing or recurrent airway obstruction (RAO).^8^, ^9^ In equine RAO, the exposure to allergens from hay and straw dust and induced symptoms are associated with increased plasma histamine levels^10^; however, at least in case of the fungus *Aspergillus*, in vitro IgE diagnosis and intradermal IgE levels do not support IgE‐mediated mechanisms.^11^ In general, involvement of IgE‐mediated mechanisms in RAO is still controversial.

The phenomenon of insect bite hypersensitivity (IBH) which especially occurs in Icelandic ponies and leads to “summer eczema” with severe pruritus, alopecia and crusting^12^ is the best investigated equine atopic disease thus far.^13^ IBH can also be associated with bronchial hyper‐reactivity^14^ and hence mirrors the human atopic syndrome characterized by cutaneous and respiratory symptoms.^15^

Interestingly, environmental allergen sources such as house dust mites (HDM), moulds^16^ and pollen^2^, ^17^ likewise elicit allergic symptoms in horses.

In addition, several food allergen sources have been described being potentially relevant for horses, including oats, wheat or corn.^18^ A major preventive and therapeutic strategy is to avoid feeding potential allergens. Hence, identification of such allergens is absolutely crucial.^19^

Diagnosis of allergic conditions and desensitization therapy in domestic animals is today performed with allergen extracts.^17^, ^20^ Intradermal challenge with extracts is a well‐established test procedure in equine allergology.^21^ However, some studies have yielded conflicting results, that is either better diagnostic accuracy than conventional serological tests^22^ or weaker reproducibility.^23^ In this context, sensitive assessment of horse serum for the presence of allergen‐specific IgE may represent a valuable diagnostic option, all the more as blood can be collected on‐site without sedation.^1^, ^24^, ^25^, ^26^, ^27^

In recent years, innovative methods for reliable allergy diagnosis have been developed. In contrast to crude allergen extracts, molecular allergy diagnosis is based on single natural or recombinant allergen molecules. Molecular allergy diagnosis has entered clinical practice in humans,^28^ allowing either approaches “from clinics to molecules” or “from molecules to clinics”.^29^ It is debated whether it might replace skin testing screenings in the future,^30^ or not.^31^

In particular, the immuno‐solid‐phase allergen chip (ISAC) has revolutionized human allergy diagnosis. The first ISAC microarray has enabled serum IgE testing on 94 allergens^32^, ^33^; the next‐generation microarray ISAC112 extended to 112 molecules is applied in today′s daily allergy diagnosis.^28^ Recently, an experimental ISAC format comprising 176 allergen molecules has been used in the European Union‐funded research project MeDALL to determine IgE sensitizations in birth cohorts.^34^ Allergen chip‐based diagnostic tools allow a risk evaluation for the grade of allergic reaction to be expected by providing individual IgE sensitization profiles. For example, in food allergy, IgE antibodies specific for very stable proteins such as 2S‐albumins or to lipid transfer proteins (LTPs) indicate a higher risk for systemic reactions in a sensitized individuals. In contrast, IgE antibodies to unstable pathogenesis‐related (PR) molecules of the PR10 family imply a lower risk. For all these reasons, it is unfortunate that component‐resolved diagnosis has not yet reached veterinary allergy diagnosis. This is one reason why today there is still a knowledge gap regarding allergen components relevant in the animals.^35^

To this aim, we expanded an allergen chip for IgE serology in animals adding 19 more molecules of potential relevance in veterinary allergy diagnosis (Table S1). We considered proteins from the following allergen sources potentially important: from other animals (albumins from rat, guinea pig and rabbit; lipocalin from mouse; alpha‐Gal; from human (uteroglobin, IgG, profilin and Hom s 2^36^), from the commensal yeast *Malassezia* associated with a number of different skin disorders such as atopic eczema,^37^, ^38^ from midges,^39^, ^40^ relevant via skin^41^, ^42^, ^43^ and from plant food.^44^

In a pilot study, using sera from clinically well‐characterized allergic horses with various symptoms and horses without clinical allergy (Table 1), we established IgE testing on ISAC131.

**Table 1:** Characterization of equine patients

Patient no.	Breed	Sex	Age (years)	Origin	Symptoms
1	Icelandic horse	M	16	Iceland▫ Switzerland	eczema, summer
2	Icelandic horse	F	16	Iceland▫ Switzerland	eczema, summer
3	Icelandic horse	M	25	Iceland▫ Switzerland	eczema, summer
4	Icelandic horse	F	>20	Iceland▫ Switzerland	eczema, summer
5	Icelandic horse	F	20	Iceland▫ Switzerland	eczema, summer
6	Icelandic horse	F	22	Iceland▫ Switzerland	eczema, summer
7	Icelandic horse	F	22	Iceland▫ Switzerland	eczema, summer
8	Icelandic horse	F	23	Iceland▫ Switzerland	eczema, summer
10	Icelandic horse	F	14	Iceland▫ Switzerland	eczema, summer
12	Icelandic horse	F	7	Iceland▫ Switzerland	eczema, summer
14	Icelandic horse	F	7	Iceland▫ Austria	eczema
15	Icelandic horse	F	11	Iceland▫ Austria	eczema
18	Icelandic horse	M	11	Iceland▫ Austria	eczema, coughing
13	Icelandic horse	F	12	Iceland▫ Austria	RAO^a^
21	Trakehner x Furioso	M (cap.)^b^	9	Austria	RAO
25	German riding pony	M (cap.)	20	Austria	RAO
37	Polish half breed	M (cap.)	14	Poland	RAO
19	Trakehner	M (cap.)	12	Austria	coughing
20	Hanoverian horse	F	17	Germany	coughing, seasonal
22	Trotter	F	12	Austria	coughing, seasonal
24	Shagya Arabian	F	17	Hungary	coughing, seasonal
26	German riding pony	F	13	Austria	coughing, seasonal
29	Holsteiner x Oldenburger	M (cap.)	3	Austria	coughing, seasonal
33	Icelandic horse	F	21	Austria	coughing, seasonal
36	Icelandic horse	M (cap.)	22	Iceland	coughing, seasonal
23	Icelandic horse	F	15	Austria	coughing, hay dust
35	Icelandic horse	M (cap.)	22	Austria	coughing, hay dust
38	Thoroughbred	M	3	Japan	urticaria
39	Thoroughbred	F	5	Japan	urticaria
40	Thoroughbred	M	4	Japan	urticaria
41	Thoroughbred	M	3	Japan	urticaria
42	Thoroughbred	F	3	Japan	urticaria
43	Thoroughbred	M	4	Japan	urticaria
44	Thoroughbred	M	3	Japan	urticaria
45	Thoroughbred	M	6	Japan	urticaria
46	Thoroughbred	F	3	Japan	urticaria
47	Thoroughbred	F	5	Japan	urticaria
48	Thoroughbred	M (cap.)	4	Japan	urticaria
49	Thoroughbred	M	3	Japan	urticaria
50	Thoroughbred	F	3	Japan	urticaria
51	Thoroughbred	F	2	Japan	urticaria
9	Icelandic horse	M	23	Germany	without
11	Icelandic horse	M	15	Germany	without
16	Icelandic horse	F	6	Austria	without
17	Icelandic horse	F	26	Austria	without
32	Icelandic horse	F	20	Austria	without
34	Icelandic horse	F	24	Austria	without
27	Hungarian Warmblood	M (cap.)	8	Hungary	without
28	Knabstrupper	F	3	Germany	without
30	Welsh Mountain Pony	M (cap.)	18	Austria	without
31	Pony x Shagya Arabian	M (cap.)	6	Austria	without

a RAO: recurrent airway obstruction.

b cap.: caponized.

## 2 MATERIALS AND METHODS

### 2.1 Horse patients

Sera from 41 horses suffering from diverse allergic symptoms and 10 horses without apparent clinical symptoms that were included (28 female, 12 entire males, 11 geldings), median age 12 years (range 2‐26 years), from Austria, Switzerland, Germany, Iceland and Japan were analysed (Table 1).

Sera were used retrospectively from other studies which were approved by the Ethics Committee of the University of Veterinary Medicine Vienna (GZ 68.205/0236‐II/3b/2011 und 68.205/0151‐II/3b/2013), by the Animal Experimental Committee of the Canton of Berne, Switzerland (No. BE 51/13) and by the Animal Care and Use Committee of Japan Racing Association (#2016‐9).

### 2.2 Design of ImmunoCAP allergen microchip ISAC131

**Figure 1:**
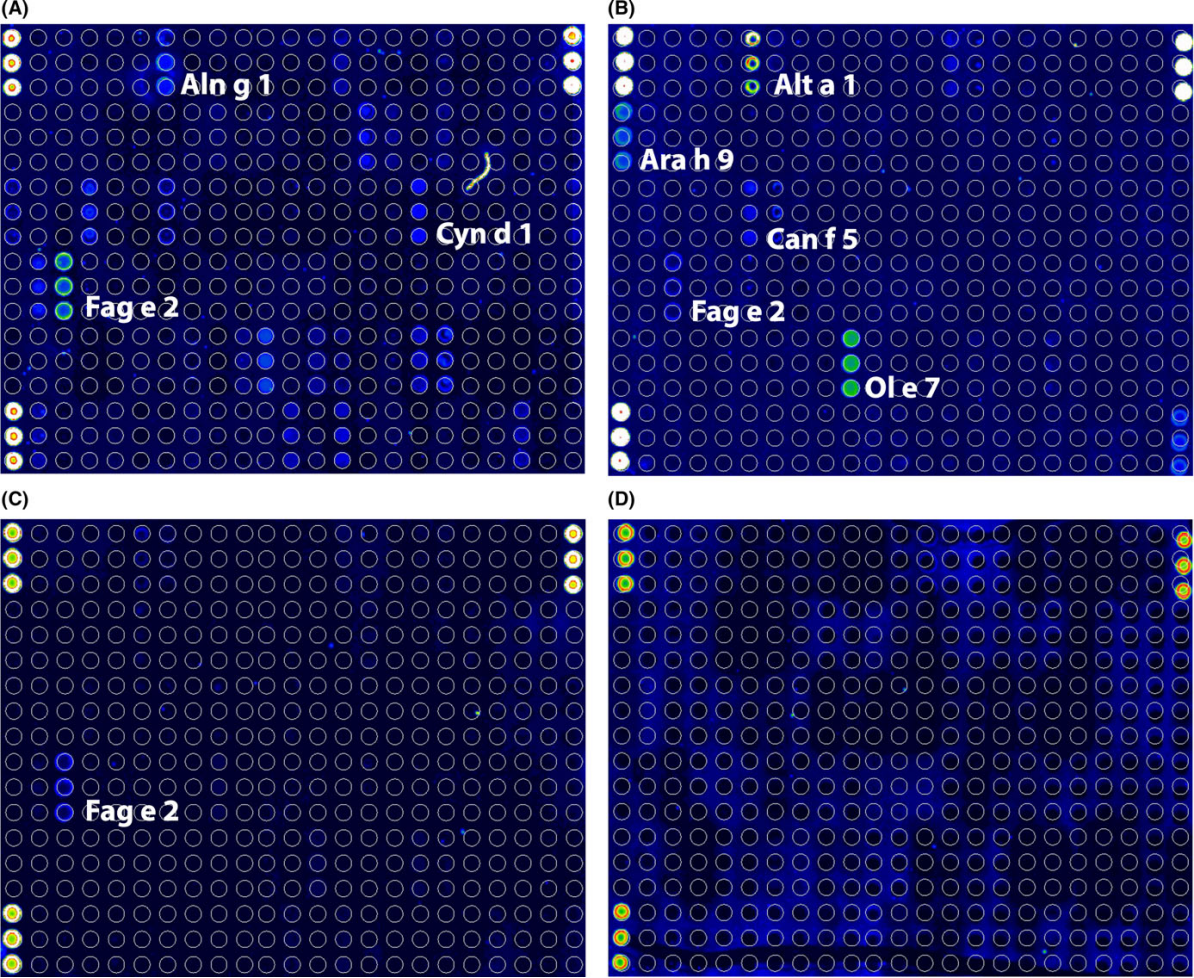
Typical laser scanning pictures resulting from testing for equine IgE on custom‐designed ISAC131. Different IgE profiles in ISAC131 are shown exemplarily for equine patients (A) no. 05; (B) no. 15; (C) no. 21; (D) no. 49 without any specific IgE to the tested allergens (clinical details in Table 1). The signal intensity corresponding to the level of specific IgE binding to 131 allergens spotted in triplicates is indicated in false colours shown in the scale below: blue, weak reactivity; green to yellow, moderate; red to white, strong IgE binding. Triplicate spots at upper left, upper right, lower left and lower right corners are spotted with calibration standards. Spotting map in Figure S1, additional allergens on ISAC131 in Table S1 [Colour figure can be viewed at http://wileyonlinelibrary.com]

In total, 131 allergen molecules (ie 112 from the commercial ISAC112 (ImmunoCAPTM ISAC, Thermo Fisher Scientific, Uppsala, Sweden), additionally 19 allergen molecules from 9 different allergen sources) were spotted using the ImmunoCAP ISAC technology as previously described^33^, rendering ISAC131 (Table S1; Figure 1A). The spotting concentration had been optimized in pre‐experiments and ranged between 0.1 ‐ 0.5 mg/mL antigen; spotted allergens were verified by specific antisera. Similarly, serum dilution strategy and detection antibody concentrations were optimized in pre‐experiments.

All detection steps were carried out according to the manufacturer's instruction, with slight modifications: Thirty μL undiluted horse serum was incubated on ISAC131 for 2 hours, and glass slides were then washed with ThermoFisher washing fluid. IgE detection (1 hour at RT) was performed 1 hour with 30 μL monoclonal mouse anti‐horse IgE antibody clone 3H10 (Bio‐Rad, Hercules, CA, USA) self‐labelled with Cy3 (Bio‐Rad, UK), diluted 1:100 in IgE dilution buffer (Thermo Fisher). After final washing, fluorescence was measured in an ISAC microchip‐reader (LUX‐Scan‐10K/A; CapitalBio Corporation, Beijing, China) with excitation at 532 nm (green laser). As the human IgE standard designed for IgE detection in human sera on ISAC112 could not be used, a work‐around was developed with the team of ThermoFisher, using an alternative positive signal with horse IgE on a spotted allergen as reference and comparing all values hereto in a semiquantitative manner. Microarray image data were analysed by MIA (Microarray Image Analysis Software, V1.2, Thermo Fisher Scientific, Uppsala, Sweden), and the ISAC standardized units (ISU) were calculated by comparing with a calibration curve (specific IgE CTRL, Thermo Fisher Scientific, Vienna, Austria, CTR02) (Figure S1B**)**. ISU values above 0.3 ISU were considered positive.

## 3 RESULTS

### 3.1 Characterization of Equine patients

A representative sample of 51 equine patients from different geographic areas, Iceland, central Europe (Austria, Germany, Switzerland, Hungary) and Japan, was assembled and horses grouped according to their clinical history (Table 1). Thirteen Icelandic horses suffered from insect bite hypersensitivity, corresponding to “summer eczema,” in one of them combined with coughing; 4 horses (an Icelandic horse, a Trakehner x Furioso cross‐breed, a German riding pony and a Polish cross‐breed) were diagnosed with equine asthma, most affected by more severe and chronic recurrent airway obstruction (RAO), except patient number 29 (3 years old) with milder inflammatory airway disease (IAD)^9^; 10 horses (4 Icelandic horses, and one each Trakehner, Hanoverian horse, Trotter, Shagya Arabian, German riding pony and a Holsteiner x Oldenburger Warmblood cross‐breed) were diagnosed with coughing, most often seasonally associated, or elicited by hay dust; all 14 Japanese Thoroughbred horses suffered from urticaria; and finally, 10 horses without allergic symptoms (6 Icelandic horses, a Hungarian Warmblood, a Knabstrupper, a Welsh Mountain Pony and a Pony‐cross‐breed), completed the panel.

### 3.2 Testing for specific equine IgE by ISAC131

Serum samples of all horses were tested on ISAC131 multiplex array (Figure S1A, B) for IgE binding to 131 allergens: 112 corresponding to commercial ISAC112, plus 19 additional allergens (Table S1). The additional allergens were selected due to potential impact for veterinary patients^35^ and comprised molecular allergens from other animals (albumins, lipocalins and alpha‐Gal), human antigens,^36^ the yeast associated with atopic dermatitis (*Malassezia sympodialis,* previously designated *Malassezia furfur*),^37^, ^38^ biting midges (*Culicoides nubeculosus*) responsible for summer eczema,^12^, ^39^, ^40^ high molecular weight enzymatic HDM allergens,^42^, ^45^, ^46^ papain^43^ and one plant allergen^44^ (Table S1).

**Figure 2:**
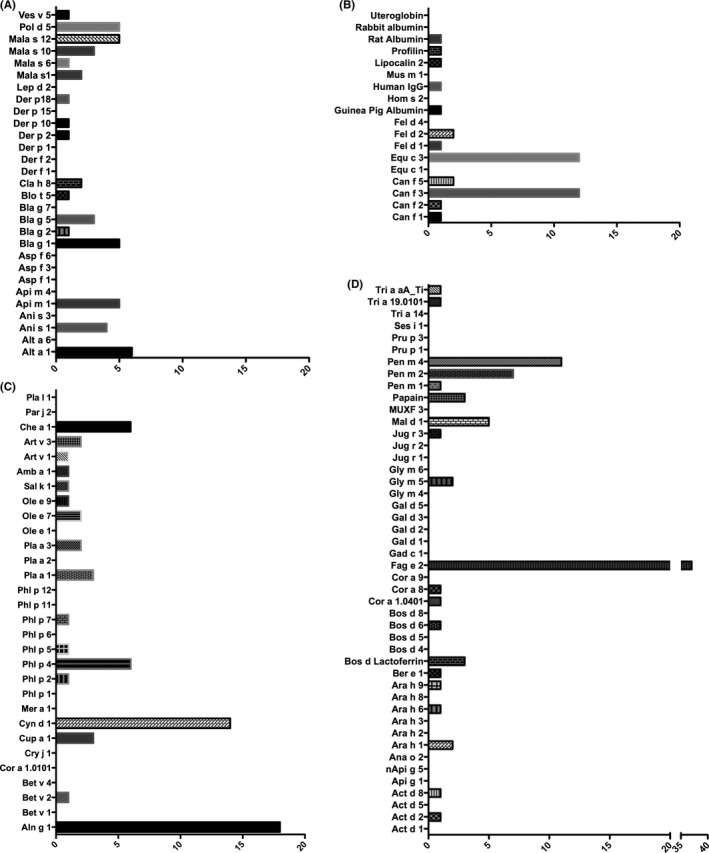
Numbers of horse patients reacting with molecular allergens spotted on custom‐designed ISAC131 microarray. Results are grouped according to allergen sources: (A) percutaneous and respiratory allergens; (B) human and animal allergens; (C) pollen allergens; and (D) food allergens. The *x*‐axis indicates the number of horses reactive to single allergen molecules in each panel; *y*‐axis: alphabetic codes of allergen molecules according to the WAO/IUIS Allergen Nomenclature (http://www.allergen.org). y‐axes in each panel: allergens spotted on ISAC131, alphabetic order; x‐axes: ISU‐E intensities

Representative results of equine IgE binding on ISAC131 (Figure 1) illustrate that IgE‐binding patterns were diverse and indicated individually differing specific IgE profiles. To extract the most important trends from the 6812 results (131 allergens × 51 sera), allergens were grouped (Figure 2), into percutaneous and respiratory sensitizers including yeast, HDM and insect‐derived molecules (Figure 2A), human and animal allergens (Figure 2B), pollen allergens (Figure 2C) and food allergens (Figure 2D). Only results accounting for over 10% of the cohort will be discussed in depth in the light of clinical symptoms. In the allergen group A (Figure 2A), 6 horses reacted specifically to fungal Alt a 1, to worm Ani s 1, cockroach Bla g 1 (mean signal intensity 1.63 ISU), earth wasp venom Pol d 5 (mean 1.52 ISU), and *M. sympodialis* Mal s 10 and Mal s 12 allergens. IgE‐binding intensities were highest in the 5 horse sera reacting to bee Api m 1 (mean 4.9 ISU).

In group B with human and animal antigens (Figure 2B), 12 horses reacted to canine Can f 3 allergen and 12 showed IgE autoreactivity to equine Equ c 1.

Among pollen allergens (Figure 2C), 18 horses reacted to alder Aln g 1 (mean 2.00 ISU), 14 to Bermuda grass Cyn d 1 (mean 3.82 ISU), 6 to timothy grass Phl p 4 (mean 1.69 ISU) and 6 to weed pollen Che a 1 (mean 1.71 ISU). In respect to food allergens (Figure 2D), 5 horses reacted to apple Mal d 1 (mean 3.24 ISU) from the PR10 family, 7 to Pen m 2 (mean 1.21 ISU) and 11 to Pen m 4 (mean 1.75 ISU) from shrimp. In total, 37 horses reacted via IgE to Fag e 2 from buckwheat (mean 5.61 ISU), among them also 7 horses from the group without symptoms.

Overall, the most abundant IgE sensitizations were seen to Fag e 2 > Cyn d 1 > Aln g 1, but we were not able to find correlations between sensitization patterns in the different clinical cohorts (Table 1).

**Figure 3:**
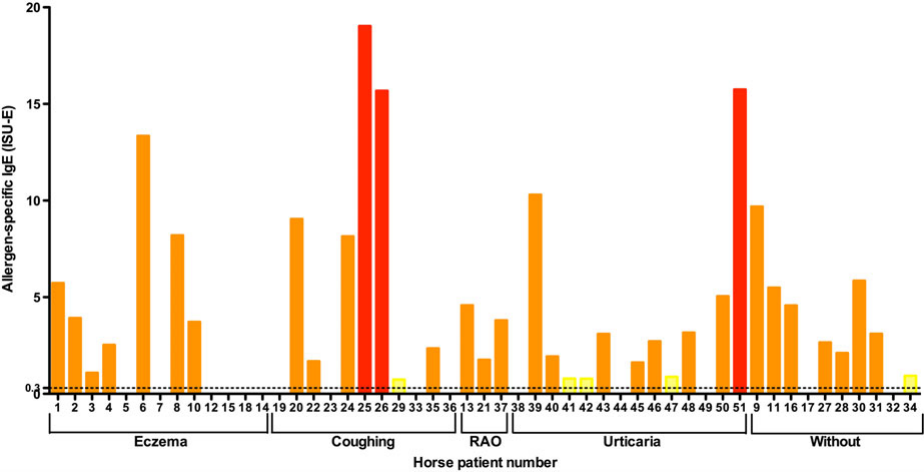
Intensities of specific IgE of single horses to the buckwheat allergen Fag e 2 in ISAC131. *X*‐axis: horses were grouped according to symptoms (Summer eczema, coughing, RAO, urticaria or without/symptoms) as described in Table 2. *Y*‐axis: levels of IgE reactivities to Fag e 2 given in ISAC standardized units **(** ISU‐E), values <0.3 ISU were interpreted as negative (indicated by the horizontal line). Results are presented in different colours depending on IgE‐binding intensity: 0.3‐0.9 ISU‐E, yellow (weak); 1‐14.9 ISU‐E, orange (moderate); >15, ISU‐E red (strong) [Colour figure can be viewed at http://wileyonlinelibrary.com]

All clinical groups show a comparable prevalence of IgE reactivity to buckwheat allergen Fag e 2, between 62% of sera from eczema, up to 100% in RAO patients (Table 2). Highest IgE‐binding intensities above 10 ISU were observed in 1 eczema, 2 coughing and 1 urticaria patients (Figure 3). The specificity of equine IgE binding to buckwheat and its 2S‐albumin Fag e 2 was confirmed by IgE inhibition experiments in immunoblotting (Data S1 and Figure S2).

**Table 2:** Specific IgE to the buckwheat allergen Fag e 2 on ISAC131 in horses grouped by allergic symptoms

Allergic symptom group	IgE to Fag e 2 in ISAC131 relative percentage	ISU‐E^a^ to Fag e 2 in ISAC131 mean ±‐STDEV
Eczema (n = 13)	61.5% (8/13)	5.5 ± 3.8
RAO^b^ (n = 4)	100% (4/4)	3.4 ± 1.5
Coughing (n = 10)	45.5% (7/11)	8.1 ± 7.1
Urticaria (n = 14)	81.4% (11/14)	4.2 ± 4.7
None (n = 10)	70% (8/10)	4.3 ± 2.8

a ISU‐E, ISAC standardized units for IgE determination, cut‐off 0,3

b RAO*,* recurrent airway obstruction.

## 4 DISCUSSION

Little is known about possible allergen sources for horses and especially about the responsible allergen molecules,^35^ including food allergy,^3^ except in summer eczema, where IgE to *Culicoides* allergens play an important role.^40^ While intradermal tests with crude Culicoides whole body often results in positive intradermal test reaction in clinically healthy horses, the use of recombinant Culicoides allergens allows a much more specific diagnosis of summer eczema,^47^ in clinically healthy, but sensitized horses.^48^

We designed the ISAC131 multiplex microarray and tested the IgE binding to 131 allergens using sera from 51 horses from different breeds and different countries of origin (Table 1). Equine total serum IgE levels are approximately 3 logs higher than in humans and did previously not discriminate allergic from healthy horses.^49^, ^50^ We also found specific IgE in the group of horses without allergic symptoms, which we interpret as clinically inapparent sensitizations.^49^ The higher IgE levels in serum of horses could have caused high background levels in ISAC131 which in three cases impeded evaluation. Furthermore, especially high IgG concentrations and their possible cross‐binding to the allergen,^51^ or competition among the multiplexed allergens for such cross‐reacting IgE could also influence the signal quality, especially at lower IgE levels.^52^

In general, the ISAC131 results (Figures 2 and 3) appropriately reflected the known susceptibility of horses to tree and grass pollen.^2^, ^5^ Interestingly, the major alder pollen allergen Aln g 1, but not Bet v 1 from birch pollen, was identified as a major respiratory sensitizer in 18 cases. Both pollen major allergens from the botanic species *Fagales* belong to the PR‐10 family, a protein family with innate immune function in plants.^53^ They are able to ignite Th2 immune responses in humans and animals by their ligand‐binding capacity.^54^ PR‐10 molecules are highly cross‐reactive and can sensitize human atopic individuals; in humans, this is usually dominated by IgE responses to the birch major allergen Bet v 1,^55^ at least in Middle and Northern Europe. We speculate that possibly around paddocks and often associated ponds, alders may be most prominent and, therefore, represent the primary sensitizing allergen source. This theory is in conflict with a recent report that for human allergics Bet v 1 is the leading allergen also in the birch‐free Mediterranean area.^56^ In 2 of the 4 horses reacting via IgE to PR10 allergen Mal d 1 from apple, co‐sensitization to Aln g 1 could be found. To this end, it has not been shown that horses may develop oral allergy syndrome, which in humans is a common clinical problem due to sensitization to PR‐10 allergens, also sporadically reported to occur in companion animals other than horses.^2^

The second most abundant sensitization was found to Bermuda grass allergen Cyn d 1 in 14 of the 51 horses investigated. As in the case of tree pollen, a great degree of IgE cross‐reactivity is elicited by grass pollen, with group 1 and group 5 pollen allergens being most important in humans. Cyn d 1 belongs to group 1 grass pollen allergens, but no cross‐reactivity was observed with Phl p 1 from timothy grass. This could be due to grass seeding strategies on paddocks, which is a hotly debated topic among horse owners. Natural (n) Cyn d 1, nPhl p 4, nApi g 5, nCup a 1 and MUXF3 on ISAC131, expresses cross‐reactive carbohydrate determinants (CCDs) which principally could lead to nonspecific IgE binding.^57^ However, only in one horse positive for Cyn d 1, simultaneous IgE reactivity could be detected to Phl p 4 and/or Phl p 1. We consider thus anti‐Cyn d 1 IgE to be specific and non‐CCD dependent, at least in the cohort of our pilot study.

In addition to the animal allergens on commercial ISAC112, we added animal and human antigens on ISAC131, however, without revealing any significant sensitization in the investigated horse cohorts. The most frequent sensitizer was Can f 3, serum albumin from dog, whereas in only one horse IgE to Can f 5 was found, suggesting exposure to a male dog.^58^ The IgE reactivities were in most incidences paired with serological IgE cross‐reactivity to Equ c 1, the equine lipocalin. We did not find IgE to cat, rabbit, rat or mouse lipocalins.

House dust mites (HDM) are abundant in households, transported by furry animals and involved in initiation and perpetuation of atopic eczema and asthma. Their enzymatic potency is involved in inflammation, while indispensable for specific sensitization.^43^ Recently, it was shown that HDM induce intelectin in epithelia,^59^ leading to subsequent IL‐25, IL‐33, and TSLP upregulation and Th2 responses. Due to the fact that dogs are frequently sensitized to high molecular weight HDM allergens, we added Der p 15 and Der p 18 on ISAC131,^41^, ^42^ but horse IgE did neither react to the human major HDM allergens Der p 1 or Der p 2 nor canine major allergens Der p 15 or Der p 18 on ISAC131. Instead, 4 horses reacted to papain, which is a functional and structural homologue of HDM allergen Der p 1 with serine protease function. Papain may be contained in shampoos and has documented percutaneous sensitization capacity.^43^ Some human HDM minor allergens, such as arginine kinase, sarcoplasmic calcium‐binding protein or haemocyanin,^60^ are not contained on ISAC112 or ISAC131. They are, however, cross‐reactive to homologous allergens in shrimp and insect allergy^61^ where they may even represent primary sensitizers.^62^ As we would like to exclude shrimps as possible constituents in equine diet, we speculate that, vice versa, IgE binding to black tiger shrimp allergens Pen m 2 and Pen m 4 in up to 12 of the investigated horse sera may indicate cross‐sensitization to corresponding allergens in HDM, or parasites, which are not yet contained on any ISAC.

There were a few indications of environmental exposure to German cockroach by IgE to Bla g 1 and 5, and little evidence for fungal exposure via IgE to *Alternaria* allergen Alt a 1 in 6 horses, and a single horse reacted with IgE to *Aspergillus* allergen Asp f 1. Hay dust may be contaminated with moulds, to which specific IgE (together with specific IgG) can be formed which previously were correlated with equine bronchitis.^25^ The ISU level to Alt a 1 in ISAC131 was highest in one RAO affected horse. However, as IgE to Alt a 1 was found in all horse groups, this does so far not allow associations to respiratory allergic symptoms.

Only sporadic sensitizations to Mala s 1, Mal s 6, 10 and 12^37^ were found. These allergens are characterized from *M. sympodialis* a lipid‐dependent yeast, part of our normal skin microbial flora, but also associated with several common skin disorders such as atopic eczema/dermatitis,^38^, ^63^, ^64^ and may be associated with food allergy.^65^ Malassezia has so far been detected on the skin of healthy horses.^66^

We failed to establish the IgE‐microarray testing for *Culicoides* allergens Cul n 1 and Cul n 2,^39^, ^40^ even in sera from the Icelandic horses suffering from summer eczema, which were previously tested positive for IgE to Cul n 1 and 2 in immunoblot. This may be explained by a methodological failure resulting in insufficient spotting of Cul n 1 and 2 to the microchip glass surface.

The most significant finding, however, was the high prevalence of IgE to Fag e 2 from buckwheat in 37 of 51 tested horses from all groups, including eczema, respiratory symptoms, urticaria and nonsymptomatic. Considering that sensitization usually correlates with exposure, the high sensitization rate is plausible. Buckwheat predominantly grows in Middle Europe and Asia (https://commons.wikimedia.org/w/index.php?curid=25570098), exactly from where our horse patients originate. The buckwheat plant (*Fagopyrum esculentum*) belongs to the mustard family and may elicit phototoxic reactions.^67^ Buckwheat plants are cultivated in main regions of Europe, Asia and North America, and its grain‐like seeds are processed to noodles, bakery products and teas,^68^ especially in the Asian cuisine. While in Europe earlier used by poor people or in “rainy days,” there is a revival of buckwheat as alternative protein source in “healthy” and vegan diets today.^69^, ^70^ It may contribute to the control of cholesterol levels in animal studies,^71^ hypertension and diabetes,^72^, ^73^ and tumour growth.^74^ Interestingly, Buckwheat is a constituent of some “horse mueslis,” or horse crackers. Unfortunately it contains Fag e 2, which belongs to the 2S‐abumin family,^75^ pepsin‐resistant food allergens^76^ with the capacity to exacerbate atopic dermatitis, elicit urticaria, angioedema and in severe cases anaphylaxis in human patients.^77^, ^78^, ^79^ Few studies describe human buckwheat allergy in Asia,^80^, ^81^ Italy,^82^, ^83^ or Britain^84^. This is the first report identifying IgE to Fag e 2 in equine sera, using ISAC131. We could confirm the specificity of anti‐Fag e 2 IgE binding to buckwheat in immunoblotting inhibition experiments. This indicates that horses may be sensitized to buckwheat, which recently has gained interest as a dietary allergen relevant for human patients^78^, ^81^ in whom buckwheat has been associated with anaphylactic reactions.^81^ However, it remains to be investigated by the veterinary discipline whether Fag e 2 indeed is responsible for any of the clinical allergic symptoms in horses.

In conclusion, we report here that IgE testing using a multiplex ISAC131 test rendered novel information of IgE profiles in a representative group of 51 horses from Europe and Japan. Even though the IgE detection to some allergens, especially *Culicoides*, needs to be improved, based on our study, testing with ISAC or other allergen microarrays could be considered as a new diagnostic opportunity for horse patients with suspected allergen sensitization and could help to develop preventive, avoidance and therapy strategies.

## CONFLICT OF INTEREST

All authors declare no conflict of interest. RV has received research grants from the Austrian Science Fund (FWF), Biomay AG, Vienna, Austria, and Viravaxx, Vienna, Austria, and serves as a consultant for the latter two companies. EJJ is shareholder of AllergyCare ‐ Allergy Diagnosis and Study Center, Vienna, Austria.

## AUTHOR CONTRIBUTIONS

LE performed ISAC tests and immunoblots and drafted the manuscript; GH helped in buckwheat extraction and immunoblots; IM, RV, SV, CR, AS and RS characterized and provided allergens for the custom‐designed ISAC131 microchip; SB, EKH, IF, KK, EM, HM, HS and AT clinically characterized allergic horses and provided equine sera and corresponding ethical approvals; IPS designed and helped in mouse immunizations; EJJ designed the study, supervised experiments and finalized the paper. All authors approved the final version of the manuscript.
