# Supplementary figures and images for: Molecular allergen profiling in horses by microarray reveals Fag e 2 from buckwheat as a frequent sensitizer
Source: Allergy. 2018 Feb 27;73(7):1436–46. doi: 10.1111/all.13417 (PMC6032949; doi:10.1111/all.13417)

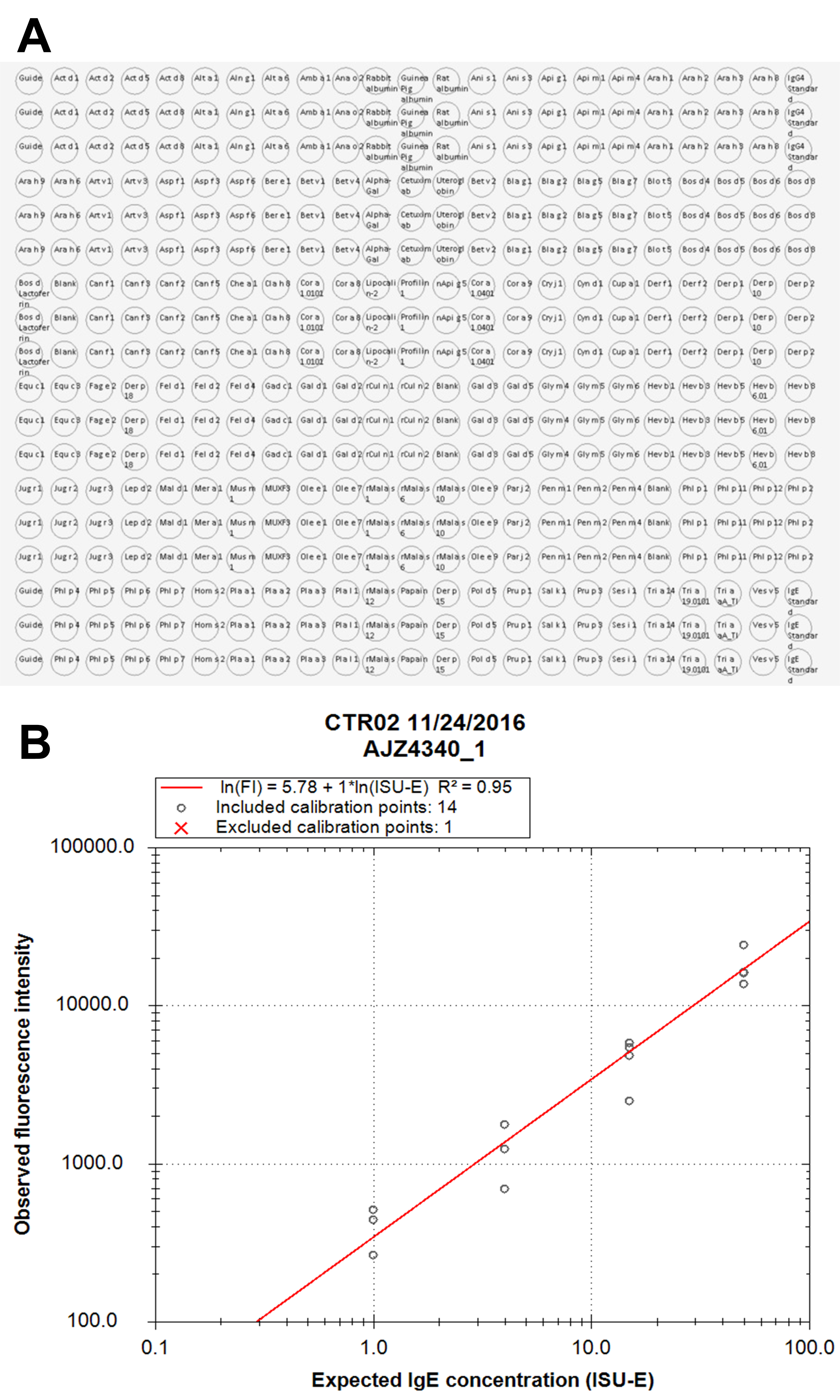

Supplement: Supplementary file 1 [file ALL-73-1436-s001.jpg]
