# Supplementary material for: Molecular allergen profiling in horses by microarray reveals Fag e 2 from buckwheat as a frequent sensitizer
Source: Allergy. 2018 Feb 27;73(7):1436–46. doi: 10.1111/all.13417 (PMC6032949; doi:10.1111/all.13417)
Supplement: Supplementary file 2 [file ALL-73-1436-s002.docx]

**Supplementary Materials**

**Supplementary Table S1.** Overview of the 19 additionally added allergen molecules on the custom-designed ISAC131 microchip.

| Category | Species | Classification | IUIS allergen name | Purchased from/ provided by |
| --- | --- | --- | --- | --- |
| Animals | Rabbit | Albumin | Rabbit albumin | Sigma |
|  | Guinea Pig | Albumin | Cav p 4 | Fitzgerald |
|  | Rat | Albumin | Rat albumin | Sigma |
|  | Mouse | Lipocalin-2 | Mus m 1 | R&D Systems |
|  |  |  | Alpha-Gal | Sigma |
| Human | Human | IgG | Human IgG | Merck |
|  | Human | Uteroglobin | Uteroglobin | R&D Systems |
|  | Human | Profilin | Profilin1 | Abcam |
|  | Human | Nascent polypeptide-associated complex alpha subunit | Hom s 2 | I. Mittermann ^(1)^ |
| Yeast | *M. sympodialis* | Unclassified | rMala s 1 | A. Scheynius ^(2)^ |
|  | *M. sympodialis* | Cyclophilin | rMala s 6 | A. Scheynius ^(3)^ |
|  | *M. sympodialis* | Heat shock protein 70 | rMala s 10 | A. Scheynius ^(4)^ |
|  | *M. sympodialis* | Glucose-methanol-choline (GMC) oxido-reductase | rMala s 12 | A. Scheynius ^(5)^ |
| Midges | Culicoides nubec. | Antigen-5 | Cul n 1 | C. Rhyner ^(6)^ |
|  | Culicoides nubec. | Hyaluronidase | Cul n 2 | C. Rhyner ^(7)^ |
| Skin enzymes | *Dermatophagoides pteronyssinus* | Chitinase-like protein | Der p 15 | Y. Resch-Marat and S. Vrtala ^(8)^ |
|  | *Dermatophagoides pteronyssinus* | Chitin-binding protein | Der p 18 | Y. Resch-Marat and S. Vrtala ^(9)^ |
|  | Papaya latex | Serin protease | Papain | Sigma ^(10)^ |
| Plant | Apium graveolens (Celery) | Berberin-bridge molecule/ FAD-containing oxidase | nApi g 5 | Lukschal et al ^(11)^ |

**References to Suppl. Table S1:**

1. Mittermann I, Reininger R, Zimmermann M, Gangl K, Reisinger J, Aichberger KJ, et al. The IgE-Reactive Autoantigen Hom s 2 Induces Damage of Respiratory Epithelial Cells and Keratinocytes via Induction of IFN-γ. *Journal of Investigative Dermatology* 2008;**128**(6):1451-1459.

2. Schmidt M, Zargari A, Holt P, Lindbom L, Hellman U, Whitley P, et al. The complete cDNA sequence and expression of the first major allergenic protein of Malassezia furfur, Mal f 1. *Eur J Biochem* 1997;**246**(1):181-185.

3. Lindborg M, Magnusson CG, Zargari A, Schmidt M, Scheynius A, Crameri R, et al. Selective cloning of allergens from the skin colonizing yeast Malassezia furfur by phage surface display technology. *J Invest Dermatol* 1999;**113**(2):156-161.

4. Andersson A, Rasool O, Schmidt M, Kodzius R, Fluckiger S, Zargari A, et al. Cloning, expression and characterization of two new IgE-binding proteins from the yeast Malassezia sympodialis with sequence similarities to heat shock proteins and manganese superoxide dismutase. *Eur J Biochem* 2004;**271**(10):1885-1894.

5. Zargari A, Selander C, Rasool O, Ghanem M, Gadda G, Crameri R, et al. Mala s 12 is a major allergen in patients with atopic eczema and has sequence similarities to the GMC oxidoreductase family. *Allergy* 2007;**62**(6):695-703.

6. Schaffartzik A, Marti E, Crameri R, Rhyner C. Cloning, production and characterization of antigen 5 like proteins from Simulium vittatum and Culicoides nubeculosus, the first cross-reactive allergen associated with equine insect bite hypersensitivity. *Veterinary Immunology and Immunopathology* 2010;**137**(1):76-83.

7. Schaffartzik A, Marti E, Torsteinsdottir S, Mellor PS, Crameri R, Rhyner C. Selective cloning, characterization, and production of the Culicoides nubeculosus salivary gland allergen repertoire associated with equine insect bite hypersensitivity. *Veterinary Immunology and Immunopathology* 2011;**139**(2):200-209.

8. O'Neil SE, Heinrich TK, Hales BJ, Hazell LA, Holt DC, Fischer K, et al. The chitinase allergens Der p 15 and Der p 18 from Dermatophagoides pteronyssinus. *Clinical & Experimental Allergy* 2006;**36**(6):831-839.

9. Resch Y, Blatt K, Malkus U, Fercher C, Swoboda I, Focke-Tejkl M, et al. Molecular, Structural and Immunological Characterization of Der p 18, a Chitinase-Like House Dust Mite Allergen. *PLoS ONE* 2016;**11**(8):e0160641.

10. Stremnitzer C, Manzano-Szalai K, Willensdorfer A, Starkl P, Pieper M, Konig P, et al. Papain Degrades Tight Junction Proteins of Human Keratinocytes In Vitro and Sensitizes C57BL/6 Mice via the Skin Independent of its Enzymatic Activity or TLR4 Activation. *J Invest Dermatol* 2015;**135**(7):1790-1800.

11. Lukschal A, Wallmann J, Bublin M, Hofstetter G, Mothes-Luksch N, Breiteneder H, et al. Mimotopes for Api g 5, a Relevant Cross-reactive Allergen, in the Celery-Mugwort-Birch-Spice Syndrome. *Allergy, Asthma & Immunology Research* 2016;**8**(2):124-131.

12. Tanaka K, Matsumoto K, Akasawa A, Nakajima T, Nagasu T, Iikura Y, et al. Pepsin-resistant 16-kD buckwheat protein is associated with immediate hypersensitivity reaction in patients with buckwheat allergy. *Int Arch Allergy Immunol* 2002;**129**(1):49-56.

13. Satoh R, Koyano S, Takagi K, Nakamura R, Teshima R, Sawada J. Immunological characterization and mutational analysis of the recombinant protein BWp16, a major allergen in buckwheat. *Biol Pharm Bull* 2008;**31**(6):1079-1085.

**Supplementary Figure S1.**

Overview of spotting map and calculation of calibration curve.

**A)** Overview of all allergen molecules spotted in triplicates on the custom designed ISAC131 chip. The 19 additionally spotted allergens are listed in **Suppl. Table S1**; the other allergens correspond to those on the commercial ISAC112 (complete list see 99356.05-2015-Product-Catalog-121914.pdf, which can be retrieved from http://www.thermofisher.com).

**B)** Calibration curve for semiquantitative analysis of IgE. ISAC131 was processed as described in the Materials and Methods section, calibrated with calibration fluid, followed by undiluted fluorescence-labeled anti-human IgE (both by Thermo Fisher Scientific, Vienna, Austria) according to the manufacturer´s description, and entitled „CTRL02“, acquired on November 24, 2016. The calibration curve was built by MIA® software depending on the criteria that a minimum of 80% of 15 calibration points render positive signal intensities, as follows: **1.0 ISU** for: Bet v 1, Der p 2, Ole e 1, Gal d 1; **4.0 ISU:** Art v 1, Fel d 1, Phl p 1; **15 ISU:** Amb a 1, Can f 1, Der p 1, Gal d 2; **50 ISU:** Can f 2, Can f 5, Phl p 5, Pru p 3. Semiquantitative analysis of allergen-specific equine IgE expressed in ISU (ISU-E) was done using the depicted calibration curve.


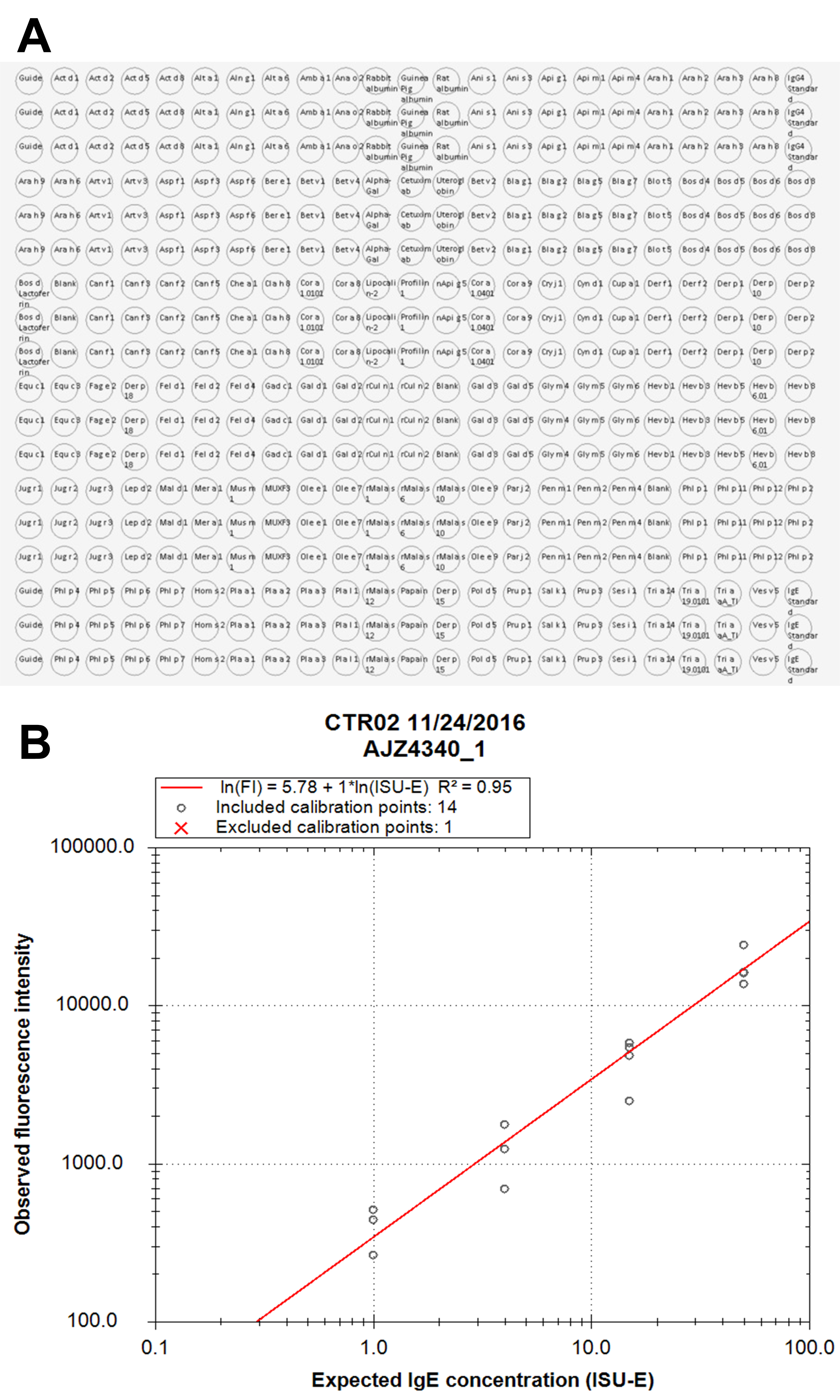


**Suppl. Figure S2**

**
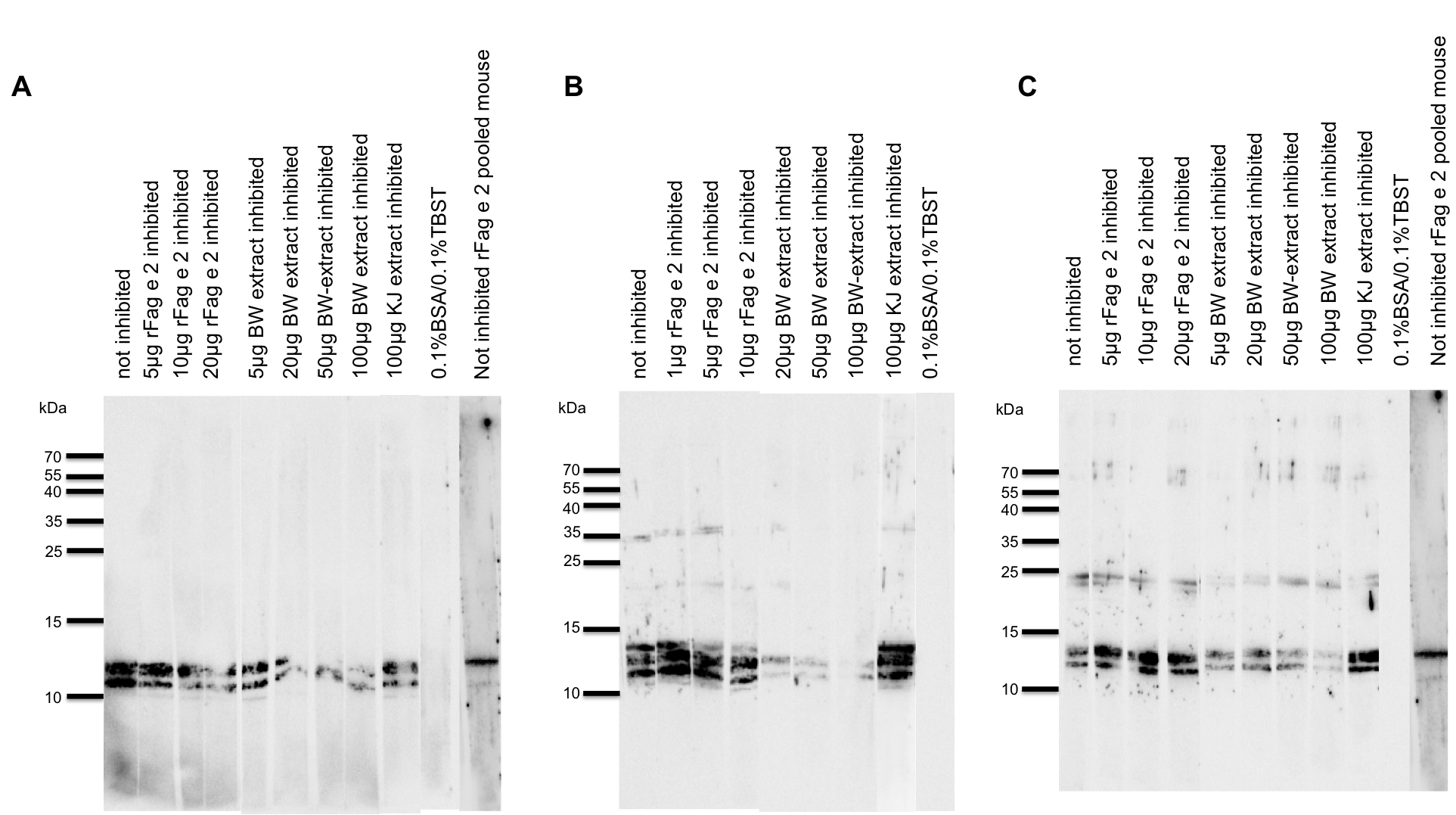
**

**Legend to Suppl. Figure S2.** Immunoblot inhibition of IgE binding using rFag e 2 and buckwheat extract in 3 selected horses, A) no. 25, B) no. 39, and C) no. 43 (see Table 1). Different dilutions of buckwheat extract (100 µg, 50 µg, 20 µg and 5 µg) or of rFag e 2 (20 µg, 10 µg, 5 µg and 1 µg), and for control codfish extract (100 µg) were used for pre-incubation of diluted horse sera. Last lane in A) and C): Blots tested with a serum pool of mice immunized with rFag e 2.

**Materials and Methods to Suppl. Fig. S2**

*Buckwheat extract and Fag e 2 protein*

Dried buckwheat grains from organic farming (Rapunzel, Legau, Germany) were grinded in liquid nitrogen. 50 g of the crushed buckwheat were suspended 1:10 in extraction buffer containing 86 mM NaCl and 33 mM NaHCO3 overnight at 4°C under constant shaking (12). After multiple centrifugation steps at 12.000 g for 30 min the extract was filtered. Finally, total protein concentration was evaluated using bicinchoninic acid assay (BCA) as recommended by the manufacturer (Pierce BCA Protein Assay Kit, Thermo Scientific, Rockford, IL, USA). Recombinant buckwheat protein rFag e 2 (BWp16) was kindly provided by Rie Satho, produced as described previously (13).

*IgE Immunoblot inhibition*

Buckwheat extract was loaded and separated under reducing conditions in 18% SDS-PAGE (Sodium dodecyl sulphate-polyacrylamide gel electrophoresis) gels (10 µg/lane) using prestained molecular weight markers (PageRuler Prestained protein ladder, Thermo Fisher Scientific), and blotted onto nitrocellulose (GE Healthcare, UK). Sera of 3 selected horse patients (no. 25, 39, 43, see Table 1) were diluted 1:100 in TBS and pre-incubated with buckwheat extract (100 µg; 50 µg; 20 µg), rFag e 2 (20 µg; 10 µg; 5 µg, 1 µg) and as a negative control with codfish extract (100 µg), over 24 h at 4°C. Saturation of the nitrocellulose membrane and all antibody dilutions were done with TRIS-buffered saline, 0.1% Tween-20 (TBST)/1% BSA (bovine serum albumin). Single blot strips were incubated with pre-incubated horse sera overnight at 4°C under constant shaking. After washing, buckwheat-specific IgE was detected using mouse anti-horse IgE (Bio-Rad, Hercules, CA, US) for 1 h at RT. Again after washing, bound IgE was detected with horseradish peroxidase (HRP) conjugated goat anti-mouse IgG (Bethyl, Montgomery, United States), for 1 h at RT. The reaction was developed by enhanced chemiluminescence (ECL) substrate (Clarity Western ECL, Bio-Rad, UK) according to the manufacturer´s instructions.

*Generation of Fag e 2-specific mouse sera*

BALB/c mice (n=5/group) were immunized 3 times in 2-weekly intervals intraperitoneally with rFag e 2 (5 µg/mouse/shot in 50 µl sterile PBS), using 100 µl Al(OH)3 (Alu-Gel-S, SERVA, Heidelberg, Germany) as adjuvant (EK number BMWF-66.009/0133-WF/V/3b/2016). Serum was taken before and 7 days after the last immunization. Sera were pooled to equal parts, diluted 1:40 and incubated on blotted in-house buckwheat extract. Bound mouse IgG was detected by goat anti-mouse IgG-Fc Fragment-HRP (Bethyl; Montgomery, TX, USA), 1:2500, and detected with chemiluminescence (ECL) substrate (Clarity Western ECL, Bio-Rad, UK) according to manufacturer´s instructions.

**Results to Suppl. Fig. S2**

**IgE-immunoblot inhibition using buckwheat allergens**

To confirm the relevance of buckwheat as an allergen source and the equine IgE reactivities on a different solid phase than ISAC, buckwheat grains were extracted, separated in SDS-PAGE and blotted to nitrocellulose (**Suppl.** **Figure S2**).

Coomassie stain showed that at the molecular mass of rFag e 2, 11-12 kDa, a double/triple band could be observed. rFag e 2 in blotted buckwheat extract was identified as a single band at 12 kDa by anti-Fag e 2 specific mouse IgG from mice immunized with rFag e 2, while equine IgE reacted to a double/triple band at 11-12 kDa (**Suppl. Figure S2**). Sera were selected from one equine patient with high anti-Fag e 2 IgE reactivity (No. 25), one with intermediate (No. 39), and one with low reactivity (No. 43). In 2 of the 3 selected sera, dose-dependent IgE-inhibition to the highest rFag e 2 band could be achieved with rFag e 2, while all 3 bands could be extinguished by buckwheat, but not by codfish extract as control. The data suggest that equine IgE reactivity to rFag e 2 identified by ISAC131 is indeed specific to buckwheat, but directed against several Fag e 2 isoforms.
